# TDP-43 Vasculopathy in the Spinal Cord in Sporadic Amyotrophic Lateral Sclerosis (sALS) and Frontal Cortex in sALS/FTLD-TDP

**DOI:** 10.1093/jnen/nlaa162

**Published:** 2021-01-09

**Authors:** Isidro Ferrer, Pol Andrés-Benito, Margarita Carmona, Abdelilah Assialioui, Mónica Povedano

**Affiliations:** 1 From the Department of Pathology and Experimental Therapeutics, University of Barcelona, L’Hospitalet de Llobregat, Barcelona, Spain; 2 Biomedical Network Research Center on Neurodegenerative Diseases (CIBERNED), Institute Carlos III, L’Hospitalet de Llobregat, Barcelona, Spain; 3 Bellvitge Biomedical Research Institute (IDIBELL), L’Hospitalet de Llobregat, Barcelona, Spain; 4 Institute of Neurosciences, University of Barcelona, Barcelona, Spain; 5 Neuropathology, Pathologic Anatomy Service, Bellvitge University Hospital, L’Hospitalet de Llobregat, Barcelona, Spain; 6 Functional Unit of Amyotrophic Lateral Sclerosis (UFELA), Service of Neurology, Bellvitge University Hospital, L’Hospitalet de Llobregat, Barcelona, Spain; 7 International Initiative for Treatment and Research Initiative to Cure ALS (TRICALS), Utrecht, The Netherlands

**Keywords:** Amyotrophic lateral sclerosis, Blood vessels, Endothelial cells, Frontotemporal lobar degeneration, Pericytes, TDP-43

## Abstract

Sporadic amyotrophic lateral sclerosis (sALS) and FTLD-TDP are neurodegenerative diseases within the spectrum of TDP-43 proteinopathies. Since abnormal blood vessels and altered blood-brain barrier have been described in sALS, we wanted to know whether TDP-43 pathology also occurs in blood vessels in sALS/FTLD-TDP. TDP-43 deposits were identified in association with small blood vessels of the spinal cord in 7 of 14 cases of sALS and in small blood vessels of frontal cortex area 8 in 6 of 11 FTLD-TDP and sALS cases, one of them carrying a *GRN* mutation. This was achieved using single and double-labeling immunohistochemistry, and double-labeling immunofluorescence and confocal microscopy. In the sALS spinal cord, P-TDP43 Ser403-404 deposits were elongated and parallel to the lumen, whereas others were granular, seldom forming clusters. In the frontal cortex, the inclusions were granular, or elongated and parallel to the lumen, or forming small globules within or in the external surface of the blood vessel wall. Other deposits were localized in the perivascular space. The present findings are in line with previous observations of TDP-43 vasculopathy in a subset of FTLD-TDP cases and identify this pathology in the spinal cord and frontal cortex in a subset of cases within the sALS/FTLD-TDP spectrum.

## INTRODUCTION

Sporadic amyotrophic lateral sclerosis (sALS) and most cases of frontotemporal lobar degeneration with ubiquitin-positive inclusions (FTLD-U) are considered to be within the same spectrum (1). This is supported by the identification of trans-activation response element (TAR) DNA binding protein-43 (TDP-43), encoded by *TARDBP* gene, as the major pathological protein in the inclusions of FTLD-U (hereinafter FTLD-TDP) with or without motor neuron disease, and in sALS (2). Aggregates of TDP-43 are identified in multiple brain areas in sALS and in FTLD-TDP (3–9).

In sALS and FTLD-TDP, loss of nuclear TDP-43 is accompanied by the formation of pathological aggregates containing phosphorylated TDP-43 (P-TDP-43) in the cytoplasm of neurons and glial cells, and in neuronal processes (2, 10, 11). In sALS, the inclusions are not restricted to the spinal cord, motor nuclei of the brainstem, or frontal and temporal cortices, but are also present in other brain regions, such as the hippocampus (7, 8, 12). Neuronal cytoplasmic TDP-43-immunoreactive inclusions in sALS have variable morphology; they may be skein-like, round hyaline, spicular, dot-like, fine granular, punctuate granular, thread-like, and perinuclear. Glial cytoplasmic inclusions (GCIs) occur in oligodendrocytes (2, 13, 14). In FTLD-TDP, TDP-43-immunoreactive aggregates are neuronal cytoplasmic inclusions (NCIs), thin and thick dystrophic neurites (DNs), and neuronal intranuclear inclusions (NIIs), in addition to GCIs (3). TDP-43 inclusions are found in the neocortex, hippocampus, dentate gyrus, and other brain regions (15–18). Transitional aggregates can be seen in sALS and FTLD-TDP (3, 19–22). The type and distribution of TDP-43 inclusions has permitted an instrumental classification of FTLD-TDP into types A, B, C, and D (15–18), as detailed below. Mutations in TDP-43 are causative of some cases of familial ALS (23–28); a few familial FTLD-TDP cases are linked to mutations in *TARDBP* (29–32).

Abnormalities in the wall of blood vessels, and altered blood-brain barrier (BBB) and SCBB, occur in sALS. Alterations affect the endothelial cells, pericytes, thigh junctions, matrix metalloproteinases, transport systems, aquaporin 4, free radicals, cytokines, and VEGF receptors, among others (33–40). A major consequence is the impairment of the BBB and the blood-spinal cord barrier (BSCB) (35, 36, 41–44). Similar alterations are seen in the blood vessels of the spinal cord in TDP-43 conditional knockout mice (45). Moreover, loss of TDP-43 in zebrafish produces axonal degeneration of motor neurons, muscular fiber degeneration, reduced blood circulation, and miss-patterning of blood vessels (46). BBB dysfunction in sALS also depends on factors linked to the CSF and peripheral blood (47–52).

Little information is available on blood vessels in FTLD-TDP, except for microbleeds (53). However, TDP-43 microvasculopathy has been described in 3 cases with familial FTLD-TDP type A, one case with familial Lewy body disease, and one case with Perry syndrome, both with accompanying TDP-43 proteinopathy; TDP-43 vasculopathy is rarely encountered in the cerebral cortex in TDP-43 types B and C (54, 55). Using immunoelectron microscopy, vascular TDP-43 deposits were identified as astrocytic end-feet with abnormal TDP-43 fibrillary inclusions, although many of them were enclosed in the capillary basal lamina. Since this important observation may represent a link between microvascular abnormalities within the FTLD-TDP/sALS spectrum and the best-known TDP-43 pathology affecting neurons, neurites, and oligodendroglial inclusions, the present study is geared to gaining knowledge about TDP-43 inclusions in association with blood vessels in the spinal cord and frontal cortex area 8 of sALS, and in frontal cortex area 8 of FTLD-TDP cases.

## MATERIALS AND METHODS

Postmortem brain cases were obtained from the Institute of Neuropathology biobank, now a branch of the HUB-ICO-IDIBELL biobank, following the Spanish legislation (Real Decreto 1716/2011), and the approval of the local ethics committee (CEIC, Bellvitge University Hospital). The brain and spinal cord were obtained at autopsy, and selected samples were rapidly dissected, kept in labeled plastic bags, and immediately frozen and stored at –80°C for further biochemical studies, or fixed in 4% buffered formalin for no <3 weeks. Thirty selected regions of the brain and spinal cord were embedded in paraffin; 4-µm-thick sections were obtained with a sliding microtome, dewaxed, and processed for current histological and immunohistochemical methods, including hematoxylin and eosin, Luxol fast blue-Klüver Barrera, Sudan black, and periodic acid Schiff. Other sections were processed for phospho-tau (AT8), β-amyloid, α-synuclein, phospho-neurofilament medium and heavy chain, ubiquitin, TDP-43, P-TDP-43 Ser409-410, glial fibrillary acidic protein (GFAP), Iba1, and myelin basic protein immunohistochemistry, as detailed elsewhere (56). sALS cases were the same as those detailed elsewhere (57). Patients were evaluated clinically according to the main signs at onset (spinal, bulbar, and respiratory) and categorized according to disease progression as fast, expected, and slow progression depending on the survival. Fast progression included patients who survived <3 years, normal progression between 3 and 5 years, and slow progression for those still alive after 5 years. The ALS Functional Rating Scale Revised (ALS-FRS-R, version May 2015) was used in every case through the clinical course of the disease. The lumbar spinal cord and the frontal cortex area 8 were examined in 14 sALS (mean age 69.5 ± 9.4 years; 6 men and 8 women). Site at onset was spinal (n = 6: 4 men and 2 women), bulbar (n = 4 female), respiratory (n = 1, man). The site of onset was not known in 3 cases (1 man and 2 women). The duration of the disease was between 3 and 5 years thus corresponding to the expected survival. All cases presented clinical symptoms consistent with upper and lower motor neuron damage. None of them presented dementia. Nine cases needed gastrostomy in the final period. All of them needed respiratory support at the final stage. The postmortem delay was between 3 hours and 17 hours. *C9orf72* expansions and mutations in *SOD1* and *TARDP* were not found in any case. Small DNs and/or TDP-43-positive granules and/or small cytoplasmic globules in cortical neurons in the contralateral frontal cortex area 8 were observed in 8 of 14 cases but were only abundant in 3 cases.

FTLD-TDP is categorized into 4 subtypes depending on the morphology of TDP-43 inclusions, laminar distribution, and relative proportion of DNs versus NCIs (15–18). Type A shows abundant short DNs and compact oval or crescent-shaped NCIs, predominantly in layer II/III of the neocortex. Moderate numbers of granular NCIs are present in the dentate granule cells of the hippocampus. TDP-43-immunoreactive GCIs are present in the cerebral white matter, and in affected subcortical regions including the striatum, thalamus, and substantia nigra. Type B shows moderate numbers of compact or granular NCIs in both superficial and deep cortical layers with few or no pre-inclusions and delicate wispy neurites which are often more abundant in the superficial cortical laminae. Characteristic, and almost exclusive to type B, is the presence of NCIs in lower motor neurons, even in the absence of clinical features of ALS. GCIs in oligodendrocytes of the cerebral white matter, medulla, and spinal cord are common. Type C includes an abundance of tortuously long neurites, predominantly in the superficial cortical laminae, with few or no NCIs. NIIs and GCIs are uncommon. Variable numbers of NCIs are present in the hippocampus, usually with a compact round morphology. Type D shows an abundance of lentiform NIIs and a few short DNs in the neocortex, not restricted to any cortical layer, with only rare NCIs. In the present series, 7 cases of FTLD-TDP-43 were assessed corresponding to 4 type A, and 3 type C. Patients presented with behavioral variant frontotemporal dementia or with semantic dementia. Although no clinical symptoms of motor neuron disease were present, 3 cases (type A) showed TDP-43-immunoreactive inclusions in the anterior horn of the spinal cord and the motor nuclei of the brainstem similar to those seen in sALS. sFLTD-TDP cases in the present series are a subset of those reported elsewhere (58) (cases 17, 18, 19, 24, 25, 26, and 28). There were 6 men and 1 woman (age 67.4 ± 8.7 years), categorized as type A (n = 4) and type C (n = 3). An additional case was a carrier of the *GRN* mutation A303AfsX57; the female patient presented with late-onset frontotemporal dementia and had eye-shaped NIIs in addition to NCIs and DNs (59). The postmortem delay in the FTLD-TDP series was between 4 and 16 hours.

Patients with associated pathology including Alzheimer disease (excepting neurofibrillary tangle pathology stages I–II of Braak and Braak), Parkinson disease, tauopathies, vascular diseases, neoplastic diseases affecting the nervous system, metabolic syndrome, hypoxia, and prolonged axonal states, such as those occurring in intensive care units were excluded. Cases with infectious, inflammatory, and autoimmune diseases, either systemic or limited to the nervous system, were not included.

### Single TDP-43 and P-TDP-43 Immunohistochemistry, Double-Labeling Immunohistochemistry, Double-Labeling Immunofluorescence and Confocal Microscopy

Single-labeling immunohistochemistry was carried out on de-waxed sections 4-μm-thick in every case. The sections were boiled in citrate buffer to enhance antigenicity and blocked for 30 minutes at room temperature with 10% fetal bovine serum diluted in PBS. Then, the sections were incubated at 4°C overnight with the primary antibody, washed, and thereafter incubated with EnVision + system peroxidase (Dako-Agilent, Santa Clara, CA) for 30 minutes at room temperature. The peroxidase reaction was visualized with diaminobenzidine and H_2_O_2_. The immunoreactions resulted in a brown precipitate.

Double-labeling immunohistochemistry was done in 2 steps. The sections were incubated at 4°C overnight with the first primary antibody, washed and incubated with the appropriate secondary antibody liked to horseradish peroxidase (HRP); the immunoreaction was visualized with diaminobenzidine and H_2_O_2_ as a brown precipitate. Then, the sections were incubated at 4°C overnight with the second primary antibody, washed and incubated with the appropriate secondary antibody/HRP. The peroxidase reaction was visualized with diaminobenzidine, NH_4_NiSO_4_, and H_2_O_2_. The immunoreaction resulted in a blue-gray precipitate. Control of the immunostaining included omission of the primary antibody; no signal was obtained following incubation with only the secondary antibodies.

Primary antibodies were rabbit polyclonal antibodies TDP43 (G400) (3488, Cell Signaling, Leiden, The Netherlands) used at a dilution of 1:100, P-TDP43 Ser403-404 (TIP-TIP-P05, Cosmo Bio, Carlsbad, CA) diluted 1:2000; rat anti-P-TDP43 Ser409-410 antibody (MABN14, Millipore, Sigma-Aldrich, Darmstadt, Germany) diluted 1:100; and mouse monoclonal GFAP antibody (Diagnostic Biosystems, Mob064, Palex Medica, Sant Cugat, Spain) used at a dilution of 1:1000, and MCAM/CD146 antibody (LS-B10746/177120, LifeSpan Biosciences, Seattle, WA) used at a dilution of 1:100. The secondary antibodies were swine anti-rabbit immunoglobulins/HRP (P0217 Dako-Agilent, Santa Clara, CA), goat anti-mouse immunoglobulins/HRP (P0447, Dako-Agilent), and donkey anti-rat IgG (H + L)/HRP (A18739, Invitrogen-Thermo Fisher Scientific, Waltham, MA), all used at a dilution of 1:100. Combinations for double-labeling immunohistochemistry were P-TDP43 Ser403-404 and MCAM/CD146 antibody, and P-TDP43 Ser403-404 and GFAP.

Double-labeling immunofluorescence was carried out in de-waxed 4-μm-thick sections which were stained with a saturated solution of Sudan black B (Merck, Glostrup, Denmark) for 15 minutes to block the autofluorescence of lipofuscin granules present in cell bodies, and then rinsed in 70% ethanol and washed in distilled water. Some sections were incubated at 4°C overnight with anti-GFAP and anti-P-TDP43 Ser403-404 antibodies; other sections with CD68 (as a marker of macrophages; Abcam ab955, used at a dilution 1:200) and anti-P-TDP43 Ser403-404 antibodies. After washing, the sections were incubated with Alexa488 or Alexa546 (1:400, Molecular Probes, Eugene, OR) fluorescence secondary antibodies against the corresponding host species. Nuclei were stained with DR (dilution 1:2000, BioStatus, Loughborough, UK). After washing, the sections were mounted in Immuno-Fluore mounting medium (ICN Biomedicals, Irvine, CA), sealed, and dried overnight. Sections were examined with a Leica TCS-SL confocal microscope. No attempt was made to quantify blood vessels with TDP-43-immunoreactive deposits in line with the lack of studies quantifying the number of NCIs, NIIs, and DNs in FTLD-TDP cases.

## RESULTS

### Spinal Cord in sALS

**FIGURE 1.**
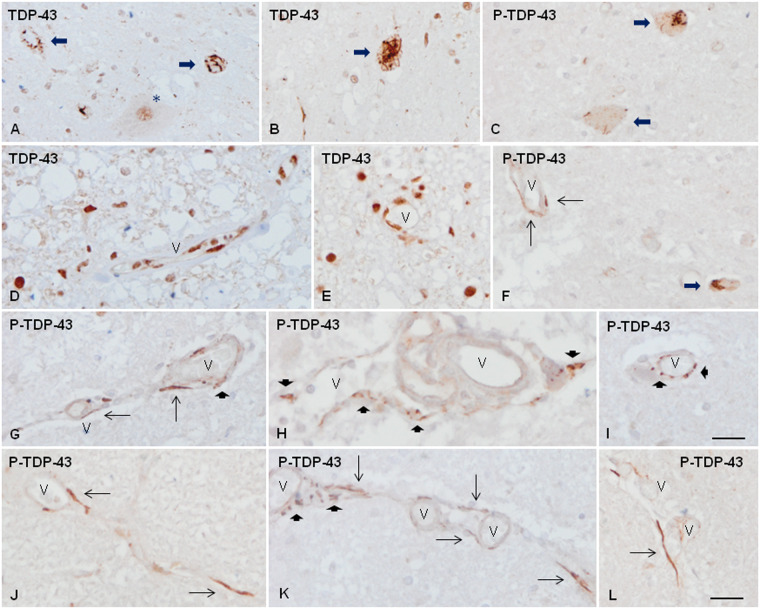
TDP-43 immunoreactivity in the anterior horn of the spinal cord in control **(D)** and sALS cases **(A–C, E-L)**. TDP-43 immunoreactivity is found in the nucleus of some remaining motor neurons **(A**, asterisk**)** and abnormal deposits in the cytoplasm of motor neurons **(A, B**, long thick arrows**)**. Abnormal neuronal TDP-43 inclusions are best distinguished with anti-P-TDP-43 antibodies **(C**, long thick arrows**)**. TDP-43 is also present in the nucleus of glial cells and the nuclei of cells of the blood vessels in controls **(D)**. In sALS, TDP-43 immunoreactivity is reduced in some nuclei of the blood vessels **(E)**. Abnormal TDP-43 deposits associated with the blood vessel walls are best seen with anti-P-TDP-43 antibodies **(F–L)**. Abnormal vascular inclusions are elongated and located parallel to the lumen (thin arrows) or forming granules (short thick arrows), often in clusters. Some abnormal deposits appear located outside the blood vessel wall **(L)**. V: blood vessel. Paraffin sections, lightly counterstained with hematoxylin, scale bars = 30 µm excepting **I**, scale bar = 25 µm.

In the normal spinal cord, TDP-43 immunoreactivity was observed in the nucleus of neurons and glial cells. In sALS, TDP-43 was preserved in the nuclei of some motor neurons of the anterior horn and the vast majority of nuclei of glial cells. TDP43 was translocated to the cytoplasm in a case-dependent number of motor neurons in sALS to form skein-like inclusions, dot-like inclusions, and granular and spicular deposits (Fig. 1A, B). Abnormal deposits were best seen with anti-P-TDP-43 antibodies which recognized only abnormal TDP-43 inclusions (Fig. 1C). In addition to neurons, isolated aberrant neurites and oligodendrocytes in the anterior horn and the white matter contained P-TDP-43 deposits. In normal spinal cord, TDP-43 was also expressed in the nucleus of cells of the blood vessel walls including capillaries of the gray and white matter, and meninges. Positive cells were in contact with the lumen or adjacent to the internal lamina (Fig. 1D). Altered TDP-43 immunoreactivity in association with blood vessels was found in 7 of 14 sALS cases. Reduced TDP-43 immunoreactivity was observed in a few vascular nuclei in the anterior horn and the white matter tracts in sALS; this reduction was selective, as TDP-43 immunoreactivity was preserved in other cells located in the vicinity (Fig. 1E). Also, abnormal TDP-43 deposits, best seen with anti-P-TDP-43 Ser403-404 antibody, occurred in association with a few small blood vessels mainly capillaries but also arterioles and venules (Fig. 1F–L). The morphology of these inclusions was variable; some of them were elongated and parallel to the lumen (Fig. 1F, G, J), whereas others were granular, often forming clusters bound to the wall of the blood vessel (Fig. 1H, I). Both types of deposits were rarely detected in a particular blood vessel along its longitudinal axis (Fig. 1K). Some elongated inclusions were outside the blood vessel wall in the vicinity of the external layer or in the perivascular space (Fig. 1L).

### Frontal Cortex Area 8 in FTLD-TDP and sALS

**FIGURE 2.**
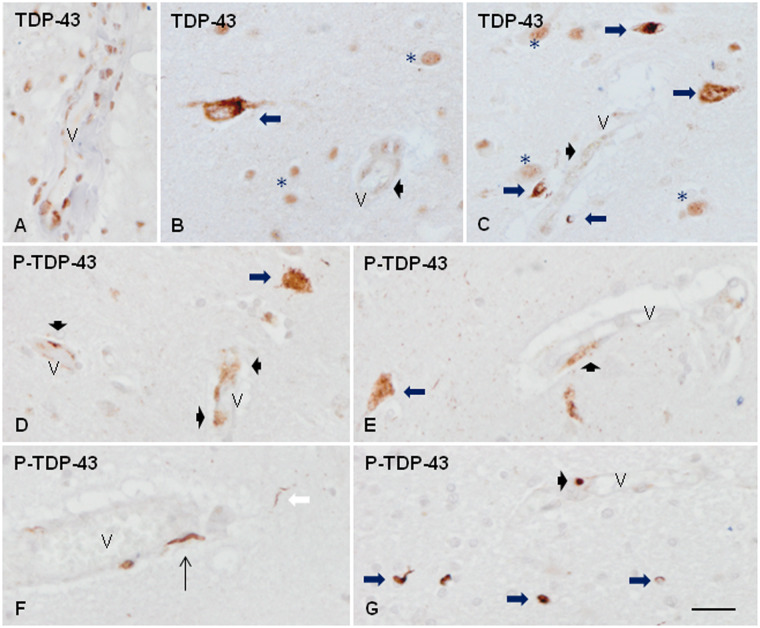
TDP-43 immunoreactivity in frontal cortex area 8 and subcortical white matter in control **(A)** and FTLD-TDP type A **(B–G)**. In the normal brain, TDP-43 immunoreactivity is found in the nuclei of cells of the blood vessel walls **(A)** in addition to the nuclei of neurons and glial cells. In FTLD-TDP, reduced nuclear TDP-43 immunoreactivity is accompanied by TDP-43-immunoreactive cytoplasmic inclusions in subpopulations of neurons and glial cells **(B, C**, long thick arrows**)**. Besides TDP-43 is decreased in the nucleus of some parietal cells of the small blood vessels and blurred TDP-43 granular deposits appeared instead **(B, C**, short thick arrows**)**. Abnormal deposits are best seen with anti-P-TDP-43 antibodies. In addition to abnormal deposits in neurons **(D, E**, thick arrows**)**, dystrophic neurites **(F**, white arrow**)**, and glial cells **(G**, long thick arrows**)**, abnormal P-TDP-43 deposits are seen in the walls of the blood vessels forming fine granular deposits **(D, E**, short thick arrows**)**, elongated threads **(F**, thin arrows**)**, and small globules **(F, G**, thin arrows**)** associated with the blood vessel wall. V: blood vessel; asterisks in **B** and **C** indicate TDP-43 immunoreactivity in the nuclei of normal neurons and glial cells. Paraffin sections, slightly counterstained with hematoxylin, scale bars = 30 µm.

In the normal frontal cortex, TDP-43 immunoreactivity was present in the nuclei of neurons, glial cells, and the nuclei of the cells of the blood vessel walls (Fig. 2A). In frontal cortex area 8 of FTLD-TDP type A, TDP-43 immunoreactivity was preserved in the majority of the nucleus of neurons, glial cells and cells of the blood vessel walls. However, TDP-43 immunoreactivity was reduced in the nucleus of a subpopulation of neurons and glial cells in which TDP-43 translocated to the cytoplasm to form NCIs with strong TDP-43 immunoreactivity in the upper and inner layers (Fig. 2B, C), and short, coma-like DNs (Fig. 2F), together with oligodendroglial inclusions in the subcortical white matter. NIIs were very rare or absent excepting in the case with the *GRN* mutation. In the frontal cortex area 8 type C and the 3 sALS cases with cortical TDP-43 pathology, TDP-43 immunoreactivity was preserved in the nucleus of the majority of neurons, glial cells and cells of the blood vessels. However, variable numbers of thick TDP-43-immunoreactive neurites (DNs) were present, mainly in the upper cortical layers and the sixth layer. NCIs were seldom observed, and in small numbers, whereas NII and glial inclusions were almost absent.

**FIGURE 3.**
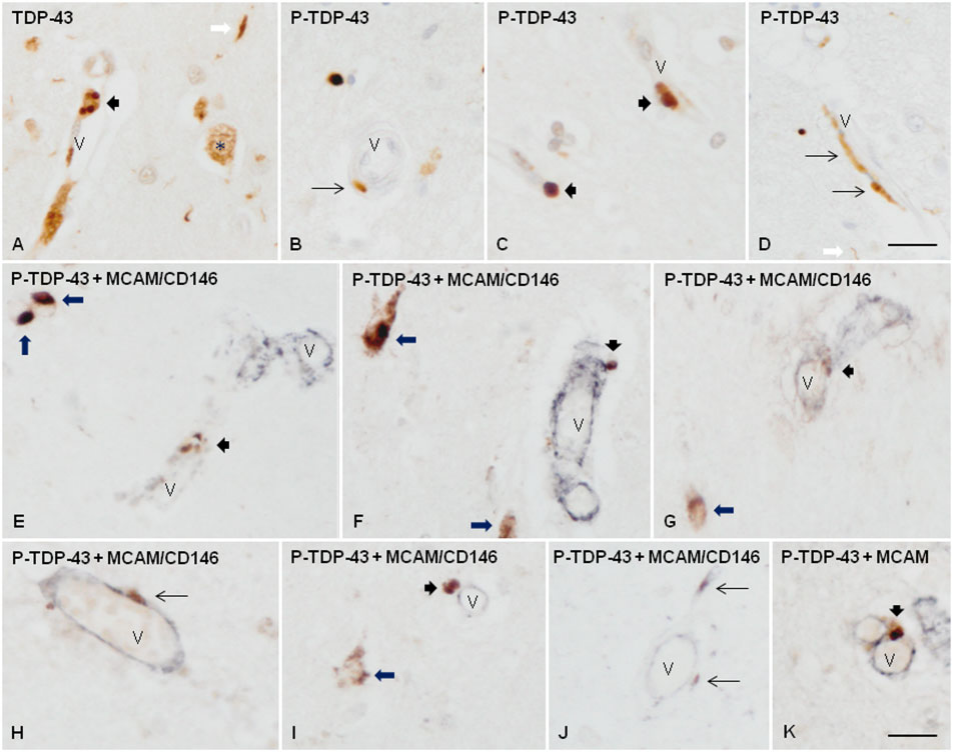
TDP-43 immunoreactivity in frontal cortex area 8 and subcortical white matter in FTLD-TDP type A. TDP-43 immunoreactivity is observed as small round inclusions **(A, C)** or as elongated or punctate inclusions **(B, D)** associated with the wall of blood vessels. Double-labeling immunohistochemistry with P-TDP-43 Ser403-404 (brown) and MCAM/CD146 (dark blue) antibodies shows the relationship of these inclusions with cells of the blood vessel walls **(E–K)**. Some of them were located within the blood vessel walls **(E, G, K)**, but others were bounded to the external surface of the blood vessels **(F, H, I, J)**. Short thick arrows indicate round inclusions, and thin arrows elongated or punctate inclusions in blood vessels; long thick arrows, neuronal TDP-43-immunoreactive cytoplasmic inclusions in neurons; white arrow, TDP-43 containing neurites; asterisk, preserved nuclear TDP-43 immunoreactivity in one neuron; V: blood vessel. Paraffin sections, lightly counterstained with hematoxylin, scale bars: **A–D **=** **30 µm; **E–K **=** **25 µm.

TDP-43 immunostaining was reduced in the nuclei of some vascular cells, and diffuse deposits appeared instead in a few blood vessels in 5 FTLD-TDP cases and one sALS case (Fig. 2B, C). Abnormal deposits in neurons and glial cells were best visualized with anti-P-TDP-43 antibodies (Fig. 2D, E, G). P-TDP-43 Ser403-404 antibody provided better staining of abnormal deposits than the antibody P-TDP-43 Ser409-410. Likewise, vascular deposits were best recognized with P-TDP-43 Ser403-404 antibodies as fine and granular (Fig. 2D, F), or elongated and parallel to the lumen (Fig. 2F), or forming small globules in the blood vessel wall (Fig. 2F, G). Besides, a few dense round TDP-43-immunoreactive bodies were seen in the neuropil, in the vicinity of, and the wall of the blood vessels in 2 FTLD-TDP types A, and in the case bearing the *GRN* mutation (Fig. 3A–D). Elongated inclusions but not round inclusions were seldom observed in association with a very few blood vessels in 2 cases of FTLD-TDP types C, and one sALS case. No inclusions associated with blood vessels were detected in the remaining FTLD-TDP/sALS cases.

### Double-Labeling Immunohistochemistry

The antibody MCAM/CD146 was used as a marker of cells of the blood vessels including endothelial cells, pericytes, and smooth muscle cells. Double-labeling immunohistochemistry with P-TDP43 Ser403-404 and MCAM/CD146 antibodies showed the presence of P-TDP-43 inclusions in 2 different compartments concerning the blood vessel walls, independently of the type of inclusion (round or elongated) in frontal cortex area 8 of FTLD-TDP; some inclusions appeared to be within the blood vessel wall, but others were located outside the blood vessel wall close to the external layer (Fig. 3E–K).

**FIGURE 4.**
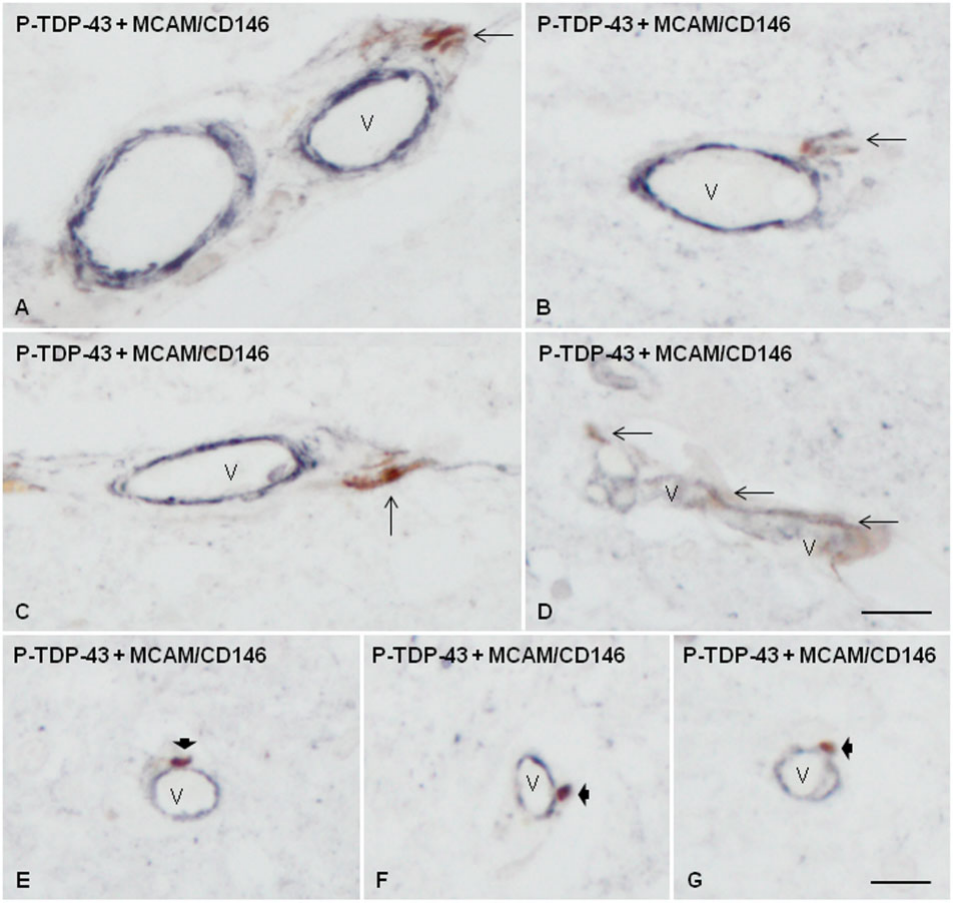
Double-labeling immunohistochemistry with antibodies P-TDP-43 Ser403-404 (brown) and MCAM/CD146 (dark blue) showing P-TDP-43-immunoreactive elongated (thin arrows) or globular (short thick arrows) inclusions in association with the blood vessels (V) in the spinal cord of sALS cases. Some elongated large inclusions were localized in the external layer or the perivascular space **(A–C)**; others in the blood vessel wall **(D)**, and others were attached to the external surface of small blood vessels **(E–G)**. Paraffin sections, scale bars = 20 µm, excepting **D**, scale bar = 25 µm.

Similarly, double-labeling immunohistochemistry in sALS cases using the same antibodies displayed P-TDP-43 inclusions outside the MCAM/CD146-immunoreactive blood vessel wall in the perivascular space (Fig. 4A–C) or in contact to the external surface of small blood vessels (Fig. 4D–G).

**FIGURE 5.**
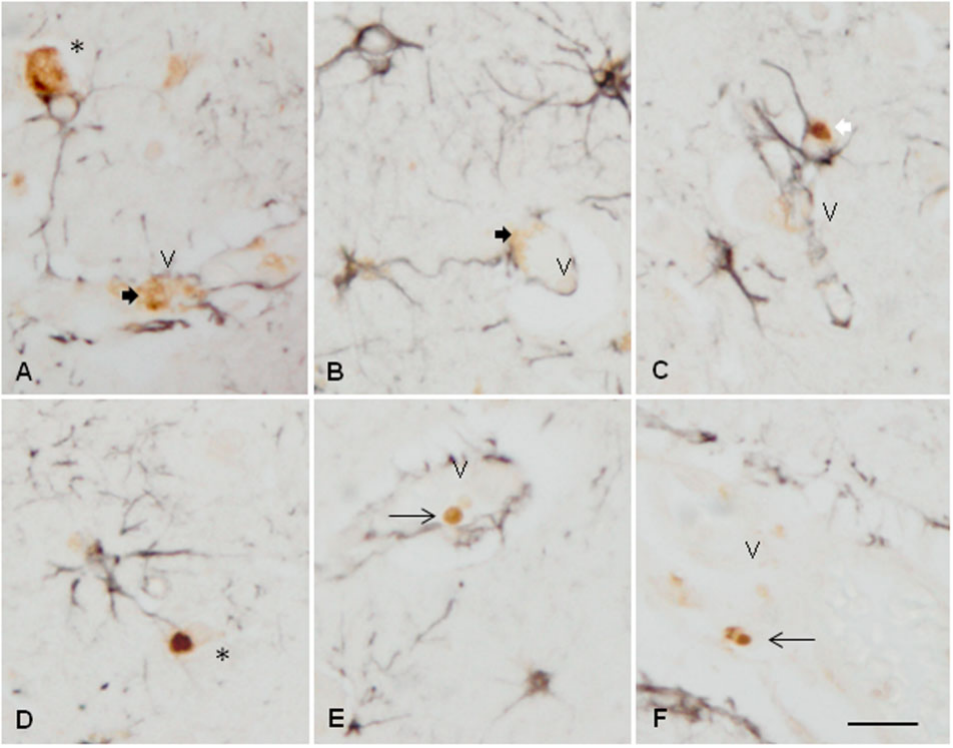
Double-labeling immunohistochemistry with antibodies P-TDP-43 Ser403-404 (brown) and GFAP (dark blue) showing that astrocytes and their processes may wrap neurons with TDP-43-immunoreactive deposits **(A, D**, asterisks**)**, and appear in contact with TDP-43-immunoreactive deposits in the blood vessel walls **(A, B)**, thick arrows. Other vascular TDP-43-immunoreactive round inclusions are separated from neighboring podocytes **(E, F**, thin arrows**)**. Only rarely, TDP-43-immunoreactive deposits appear located in the cytoplasm of astrocytes **(C**, white arrow**)**. V: blood vessel. Paraffin sections, scale bar = 30 µm.

**FIGURE 6.**
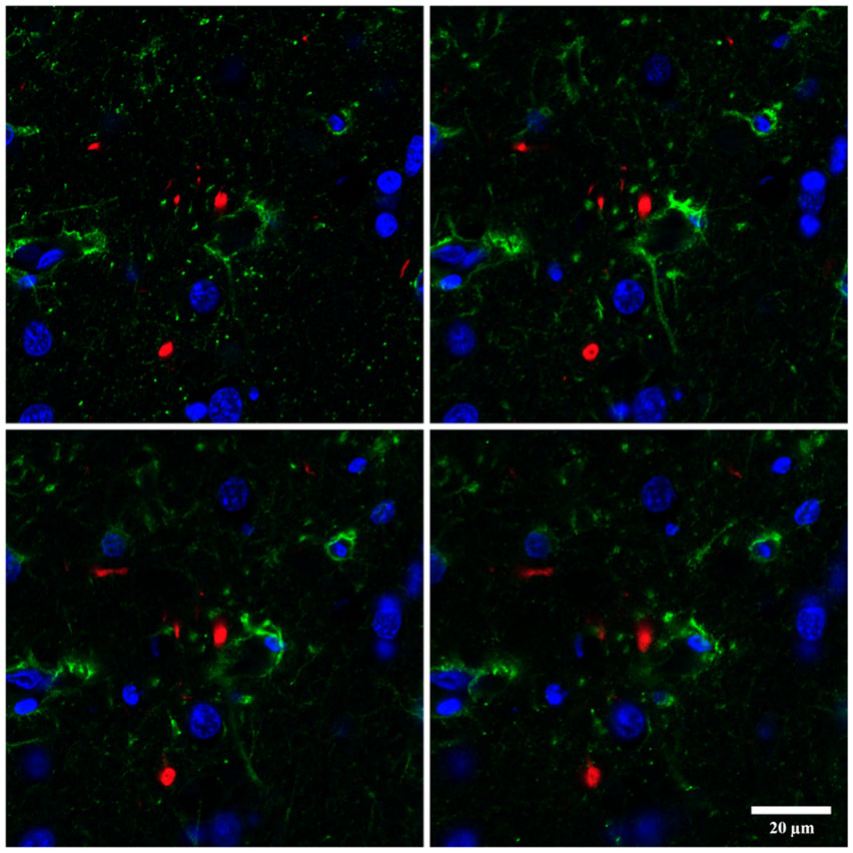
Serial images of double-labeling immunofluorescence and confocal microscopy with antibodies P-TDP-43 Ser403-404 (red) and GFAP (green) show one round inclusion close to the cytoskeleton of an astrocyte. However, the resolution of the method does not permit us to visualize whether the inclusion is within the cytoplasm of this cell. Paraffin sections, nuclei stained with DR (blue); scale bar = 20 μm.

To learn about the relationship between TDP-43-immunoreactive inclusions and astrocytes, double-labeling immunohistochemistry with P-TDP43 Ser403-404 and GFAP antibodies was carried out in the frontal cortex of cases with FTLD-TDP pathology. GFAP immunoreactivity was rarely seen in the proximity of P-TDP-43-immunoreactive deposits. In most cases, it was difficult to ascertain the relationship between some inclusions and astrocytes; this occurred when astrocyte processes wrapped small neurons with NCIs (Fig. 5A, D), and when podocytes were in proximity with TDP-43-immunoreactive vascular inclusions (Fig. 5A, B). In some instances, round TDP-43-immunoreactive vascular inclusions were not in contact with podocytes (Fig. 5E, F). Rarely, round TDP-43-immunoreactive inclusions appeared localized in the cytoplasm of astrocytes (Fig. 5C). The difficulty to verify the localization of TDP-43-immunoreactive deposits in astrocytes also occurred using serial reconstruction of sections double-labeled for immunofluorescence and examined with confocal microscopy (Fig. 6). Sections double-labeled with CD68 and TDP-43 Ser403-404 antibodies revealed no relationship between macrophages and TDP-43-immunofluorescent deposits (data not shown).

## DISCUSSION

The present study identifies TDP-43 proteinopathy in a very few blood vessels of the spinal cord in 7 of 14 sALS, in the frontal cortex area 8 in one of 3 sALS, and in the frontal cortex area 8 in 5 of 8 FTLD-TDP cases, one of them bearing a *GRN* mutation (2 type A, 2 type C and the one bearing the *GRN* mutation).

Progranulin deficiency is linked to TDP-43 pathology, including altered autophagy, TDP-43 accumulation, TDP-43 translocation, and abnormal truncation (60–63). The progranulin mutation might represent a factor of TDP-43 vulnerability in brain blood vessels. However, this factor cannot be applied to sALS and the rest of FTLD-TDP cases.

The present observations are in line with pioneering descriptions of TDP-43 vasculopathy in FTLD-TDP (55). However, the methods used in the present study are not sufficient to corroborate the astrocytic localization of at least some globular inclusions as revealed with the more precise immune-electron-microscopic approach used by Lin et al (55).

RNA processing is essential for regulated gene expression; therefore, defects at some stages of gene regulation may contribute to disease-RNA specific alterations (64–66). TDP-43 is an RNA-processing protein with roles in multiple stages of RNA regulation including RNA transcription, splicing, transport and translation, and microRNA production (67–73). TDP-43 phosphorylation and TDP-43 translocation from the nucleus to the cytoplasm, forming TDP-43 aggregates impairs TDP-43 signaling functions in the nervous system (4, 11, 17, 74–76).

Little is known about TDP-43 and blood vessels. However, the function of TDP-43 in blood vessels is probably similar to that reported in other settings including RNA transcription, RNA splicing, and protein interactions. Therefore, it may be posited that TDP-43 pathology in blood vessels may have deleterious effects in vascular homeostasis and compromise normal BBB and BSCB function in subgroups of patients with sALS/FTLD-TDP. In this line, altered blood vessels of the spinal cord are found in TDP-43 conditional knockout mice (45). Endothelial cells with abnormal mitochondria and swollen cytoplasm, pericytes containing abnormal mitochondria and disorganized materials, edematous podocytes, and splitting of the basal laminae containing degenerated organellae are found at early symptomatic stages in TDP-43 conditional knockout mice (45). Reduced blood circulation, due in part to abnormal cardiac muscle cells, and miss-patterning of blood vessels with supernumerous and hyperbranched sprouts are found in zebrafish with loss of TDP-43 (46).

The significance of perivascular and vascular TDP-43 pathology in a subset of ALS/FTLD cases is currently unknown but could be better understood through mechanistic and clinicopathological correlation studies. Not all sALS/FTLD-TDP cases contain vascular TDP-43-immunoreactive deposits, and when they are present, the number of blood vessels with positive deposits is very small. Yet, the low density of deposits could be compatible with a toxic impact on vascular cells and perivascular astrocytes.

These observations also suggest that sALS and FTLD-TDP are degenerative diseases not limited to neurons and oligodendrocytes as other cell types are affected. ALS-derived fibroblasts show cytoplasmic TDP-43 aggregation under certain experimental paradigms (77–79). Downregulated proteins specifically expressed in muscles as C-filamin, and primary muscular degeneration occur in zebrafish lacking TDP-43 (46). TDP-43 dysfunction in Drosophila causes alterations in muscle cells (80). TBPH (TDP-43 in drosophila) overexpression produces TBPH aggregates surrounding nuclei that are devoid of anti-TBPH immunolabeling suggesting that gain of function leads to the nuclear depletion of Drosophila TDP-43 in some muscle fibers (80). Abnormal TDP-43-immunoreactive inclusions occur in muscle cells in inclusion body myositis (81) and myofibrillar myopathies (82), which are considered paradigms of degenerative muscular diseases with abnormal protein aggregates, and inclusion body myopathy, Paget disease, and frontotemporal dementia (IBMPFD), which can present as a spectrum of ALS, FTLD, and myopathy with abnormal TDP-43 inclusions (83).

In conclusion, the present observations show the presence of TDP-43 vasculopathy in the cerebral cortex and spinal cord in a subset of patients within the spectrum of sALS/FTLD-TDP. Since diverse abnormalities converge in the wall of blood vessels and alter BBB and SCBB in sALS, and vascular alterations are reported in animal models showing downregulation or overexpression of TDP43 it is tempting to speculate that TDP-43 vasculopathy contribute to the altered vascular function in sALS/FTLD-TDP43 spectrum.

## Notes

This project received funding from Fundació Miquel Valls, “Retos Todos Unidos Contra la ELA” and “Proyecto DGeneracion Conexiones con Sentido” to M.P. The study was also supported by the Ministry of Economy and Competitiveness, Institute of Health Carlos III (ISCIII) (co-funded by European Regional Development Fund, ERDF, a way to build Europe): FISPI17/000809 to I.F. We thank CERCA Programme/Generalitat de Catalunya for institutional support.
